# Features of age-related response to sleep deprivation: *in vivo* experimental studies

**DOI:** 10.18632/aging.203372

**Published:** 2021-07-28

**Authors:** Maria Novozhilova, Tatiana Mishchenko, Elena Kondakova, Tatiana Lavrova, Maria Gavrish, Svetlana Aferova, Claudio Franceschi, Maria Vedunova

**Affiliations:** 1 Institute of Biology and Biomedicine, National Research Lobachevsky State University of Nizhny Novgorod, Nizhny Novgorod 603022, Russia; 2 Institute of Information Technologies, Mathematics and Mechanics (ITMM), National Research Lobachevsky State University of Nizhny Novgorod, Nizhny Novgorod 603022, Russia

**Keywords:** sleep deprivation, aging, learning ability, PLIN2, DNA methylation

## Abstract

Insomnia is currently considered one of the potential triggers of accelerated aging. The frequency of registered sleep-wake cycle complaints increases with age and correlates with the quality of life of elderly people. Nevertheless, whether insomnia is actually an age-associated process or whether it acts as an independent stress-factor that activates pathological processes, remains controversial. In this study, we analyzed the effects of long-term sleep deprivation modeling on the locomotor and orienting-exploratory activity, spatial learning abilities and working memory of C57BL/6 female mice of different ages. We also evaluated the modeled stress influence on morphological changes in brain tissue, the functional activity of the mitochondrial apparatus of nerve cells, and the level of DNA methylation and mRNA expression levels of the transcription factor HIF-1α (Hif1) and age-associated molecular marker PLIN2. Our findings point to the age-related adaptive capacity of female mice to the long-term sleep deprivation influence. For young (1.5 months) mice, the modeled sleep deprivation acts as a stress factor leading to weight loss against the background of increased food intake, the activation of animals’ locomotor and exploratory activity, their mnestic functions, and molecular and cellular adaptive processes ensuring animal resistance both to stress and risk of accelerated aging development. Sleep deprivation in adult (7-9 months) mice is accompanied by an increase in body weight against the background of active food intake, increased locomotor and exploratory activity, gross disturbances in mnestic functions, and decreased adaptive capacity of brain cells, that potentially increasing the risk of pathological reactions and neurodegenerative processes.

## INTRODUCTION

Sleep is a physiological state periodically replacing wakefulness and characterized by an absence of conscious mental activity and a significant decrease in responses to external stimuli [1–4]. Modern concepts of sleep demonstrate its crucial importance for normal functioning of the body, having a significant impact on the physical condition, emotional status, hormonal background, mnestic and cognitive human abilities [5]. Currently, it is known that sleep is a key participant of homeostatic regulation in the brain, cleansing the extracellular space from metabolic products, terminal degradation of unused synapses, preventing the amyloid proteins aggregation by activating the glial lymphatic system [6–12].

In view of rapid growth of technological progress and stress loads aggravation, the issues of sleep duration and its quality are becoming more relevant for public health.

Insomnia, including chronic insomnia, refers to stress-associated processes without clear symptoms or classical therapeutic methods for correction. It can also act as an independent stress factor, leading to disorders of a wide range of organs and systems, including the brain, blood circulation, digestion, immune functions and metabolism [13, 14]. Total sleep deprivation for 3-4 days is fatal for humans and animals [15–17]. Concerning the central nervous system, the most pronounced manifestations of insomnia are the development of neurological deficits, depression, anxiety and increased fatigue [18–22].

However, chronic sleep deprivation and circadian rhythm disturbances can induce a cumulative effect acting as a key trigger for impairments in the body's adaptive systems and the emergence of more serious pathologies [16, 23, 24]. Numerous experimental and clinical studies demonstrate that sleep quality is associated with the risk of developing schizophrenia, an early manifestation of Alzheimer's disease, Parkinson's disease and other age-related diseases of the central nervous system [3, 25–32].

Women are more likely to suffer from insomnia than men [33, 34]. At the same time, when correcting disorders caused by insomnia, higher remission rates were found among men [35]. However, the prevalence of insomnia in various forms increases in the elderly and significantly affects their quality of life [35, 36]. Natural physiological changes in circadian rhythm influence many older people to go to bed and wake up earlier; it contributes to the deterioration of sleep quality and reduction of sleep duration. A series of studies revealed that changes in the duration of the REM (rapid eye movement) sleep phase are age-dependent and show a tendency to decrease [16, 37–39]. About 50% of older people complain of difficulty falling asleep or maintaining sleep, which leads to a deterioration in the course of comorbidities and memory scores [40–45]. The data accumulated to date does not provide a clear understanding of the extent to which the effects of insomnia are age-related and whether insomnia can stimulate age-mediated neurodegeneration [46–48], which necessitates comprehensive research at different levels of the organization.

In this study, we analyzed the effects of long-term sleep deprivation modeling on the locomotor and orienting-exploratory activity as well as spatial learning abilities and working memory of C57BL/6 female mice of different ages. We also evaluated the modeled stress influence on morphological changes in brain tissue, the functional activity of the mitochondrial apparatus of nerve cells, and the level of DNA methylation and mRNA expression levels of the transcription factor HIF-1α (Hif1) and age-associated molecular marker PLIN2.

## RESULTS

**Figure 1:**
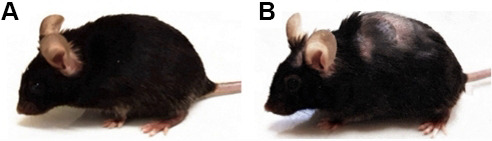
**Female C57BL/6 mice used in the experiment.** (**A**) 1.5 months; (**B**) 7-9 months.

Aging is a complex process with no clear time frame for its beginning. One of the hallmarks of aging is changes in the appearance and development of cognitive impairments. In order to analyze whether sleep deprivation launching accelerated aging processes, in our study, we used female C57BL/6 mice at different stages of the postnatal period – 1.5 (young) and 7-9 (adult) months (Figure 1). The young mice are characterized by regular fur and clear sensory-motor functions. The older generation of mice already shows a loss of fur both on the body and muzzle up to 30-40%. The fur loses its luster and turns gray. The adult mice have also exceeded the weight of young specimens (1.5 months – 19.59±0.29 g; 7-9 months – 27.38±0.36 g) and had a lower locomotor and social activity that corresponds to the previously published data [29].

Our preliminary experiments with using a flowerpot method of REM sleep deprivation by M. Jouvet [49] have shown that an increase in the time spent by a mouse on the platform for more than 12 hours led to the death of more than 80% of adults within 3-4 days. Since our study is focused on the age-related aspects of insomnia, we selected the sleep deprivation regimes at which the survival rate of adult animals was maximal (for more details please see “Methods” section). The deprivation regimes we used were close to the acute type, associated with a severe disturbance of the circadian rhythm (the development of delayed sleep phase syndrome) and the presence of negative reinforcement (the emotional factor of fear of falling into the water during sleep), inducing the development of a self-deprivation state.

### Age-related changes in the eating behavior of mice under sleep deprivation influence

Disturbances in melatonin metabolism that occur with insomnia are associated with altered eating behavior and increased food intake [50–52]. Therefore, we first compared eating behavior characteristics in mice with a regular sleep-awake cycle and sleep deprivation. The study revealed that each animal from the “Intact” and “Control” groups consumed on average 8.8±3 ml of water and 5-7 g of food per day. The total time of sleep was 10 hours a day, dividing into approximate periods of 1 hour. Despite the fact that mice are nocturnal burrowing animals and usually have a main wakefulness period at night, the sleep period did not exceed 3 hours.

Sleep deprivation modelling led to an increase in food intake by mice. Consumption of water and food in the “SD” group increased by 1.5-2 times, although the mice spent only 13 hours a day in the home cage and slept most of that time. Interestingly, the severity of the observed changes depended on the mice age. From the end of the 5th session of sleep deprivation, the adult mice (7-9 months) began to consume large quantities of food and water and quickly fell asleep in their home cages. Their activity decreased significantly, and the mice ceased to resist when we removed them from the platform. The response to nighttime signals was minimal by the end of sleep deprivation modelling. The young mice began to demonstrate the decreased activity from day 7 after the beginning of sleep deprivation, although they increased the consumption of food and water from the second day of the modeled stress.

**Figure 2:**
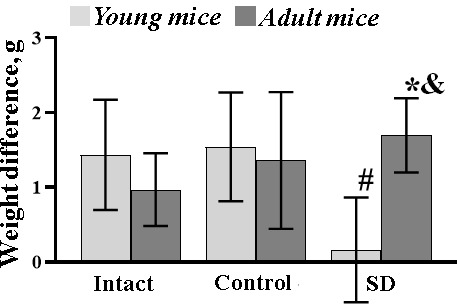
**The weight difference of female C57BL/6 mice between the start and the end of sleep deprivation modelling.** *- versus “Intact”, # - versus “Control”, p≤0.05, the Wilcoxon T-test.

Weight characteristics of mice were changed in an age-dependent manner (Figure 2). Initially, the adult mice weighed more than the young ones (Supplementary Figure 1); however, both young and adult animals from the “Intact” and “Control” groups had no significant changes in the body weight characteristics between the beginning and the end of the experiment. In contrast, sleep deprivation leads to significant weight loss in young mice after the end of the modeled stress despite their active food and water consumption. On the other hand, the adult mice gained weight. Significant weight changes were observed in the “SD adult” group relative to the “Intact” values.

### Features of locomotor and orienting-exploratory activity of mice after sleep deprivation

Analysis of animals’ behavior in the “Open Field” test showed a significant increase in locomotor activity of mice exposed to sleep deprivation modelling (Table 1). This effect was observed in both young and adult mice. The average speed crossing the arena squares in the “SD young” and “SD adult” groups was 0.45 ± 0.02 squares/s and 0.39 ± 0.02 squares/s respectively that significantly exceeded the intact and control values.

**Table 1:** Parameters of behavioral reactions of female C57BL/6 mice in the “Open field” test day after the end of sleep deprivation.

**(A) Young mice (1.5 months).**					
**Experimental groups**	**Average speed crossing the arena, squares /s**	**Number of squares passed in the arena**		**Time in the arena center, s**	**Number of peeks in holes**
		**Periphery**	**Center**		
Intact	0.26±0.03	57.6±6.0	20.0±4.3	41.3±7.14	27.33±4.33
Control	0.28±0.02	66.0±6.27	22.8±3.1	42.4±5.52	26.3±2.59
SD	0.45±0.02*#§۷	109.4±7.36*#§۷	27.0±3.26	38.4±4.35**§**	42.1±4.08*#§۷

**Table:** 

**(B) Adult mice (7-9 months).**					
**Experimental groups**	**Average speed crossing the arena, squares /s**	**Number of squares passed in the arena**		**Time in the arena center, s**	**Number of peeks in holes**
		**Periphery**	**Center**		
Intact	0.29±0.02	58.2±5.21	31.83±2.24§	60.66±7.73	26.83±5,82
Control	0.28±0.02	64.6±4.76	23.0±1.9*	61.95±8.86	24.31±2.28
SD	0.39±0.02*#§۷	86.85±4.67*#§۷&	31.85±2.94**#**	49.0±3.52	32.04±1.85**#&**

*- versus “Intact”, # - versus “Control”, § - versus “Intact” of the adjacent age group, ۷ - versus “Control” of the adjacent age group, & - versus “SD” of the adjacent age group, p≤0.05, the Wilcoxon T-test.

Moreover, the mice subjected to sleep deprivation gained increased exploratory activity. The mice of both aging groups left from the arena center and actively moved along its periphery. Of note, the young mice were more agile. The number of crossed squares in the arena periphery in the “SD young” and “SD adult” groups was 109.4±7.36 and 86.85±4.67, respectively. The young mice also had a more pronounced hole board exploratory behavior (number of peeks in holes: “SD young” 42.1±4.08, “SD adult” 32.04±1.85, p≤0.05). At the same time, the vertical motor activity was increased in adult mice compared to the young ones (the number of upright postures: “SD young” 4.3±1.0, “SD adult” 10.2±1.3, p≤0.05, Supplementary Table 1). An active search for an exit out of the arena points to an unstable emotional state and the increased anxiety level in mice. It can be assumed that such behavioral changes are associated with gradually cumulative response to long-term sleep deprivation and related to hyperactivity of the nervous system after chronic stress. Young mice respond to stress more actively, probably due to their increased adaptive capacity relative to the adult animals.

### Features of spatial learning and working memory of mice during aging and sleep deprivation

**Figure 3:**
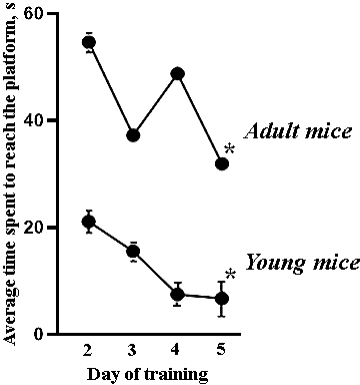
**Average time spent by female C57BL/6 mice to reach the platform during the training course in the Morris water maze.** All adult mice’s values significantly differed from those of the young mice, *- versus the second day of the training course, p≤0.05, the Wilcoxon T-test.

Our studies have shown that aging decreases the spatial learning abilities of female mice. During the training courses in the Morris water maze, the mice of 7-9 months of age spent significantly more time searching a platform, and the number of effective attempts to achieve the platform was lower compared to the 1.5 months mice (Figure 3). The young mice demonstrated fast positive dynamics of learning and predominantly used the direct searching strategy to find the platform by the end of the training course.

**Figure 4:**
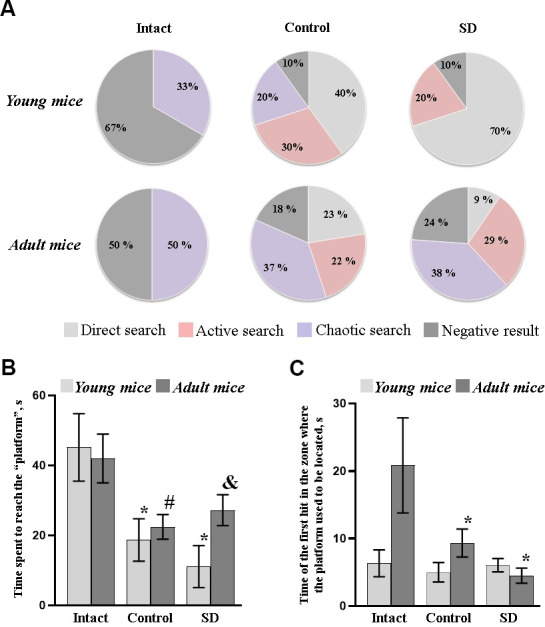
**Long-term memory retention test of female C57BL/6 mice in the Morris water maze after sleep deprivation modeling.** (**A**) Distributions of target searching strategies; (**B**) Time spent to reach the “platform”; (**C**) Time of the first hit in the zone where the platform used to be located. *- versus “Intact”, # - versus “Control”, & - versus “SD” of the adjacent age group, p≤0.05, the Wilcoxon T-test.

Long-term memory retention was assessed by testing animals in the pool without a platform. The young and adult mice from the “Intact” group who first placed in the pool had different behavioral tactics (Figure 4). The 7-9 months mice moved around the pool chaotically and showed the greatest time of the first hit to the zone where the platform used to be located. The 1.5 months mice were more active; however, they mostly moved along the thigmotaxis zone and spent more time to reach the “platform” (Figure 4A).

The young mice who previously trained in the Morris water maze (“Control”) mainly chose active (30%) and direct (40%) searching strategies, while the strategies of adults’ specimens were evenly distributed (Figure 4A).

Sleep deprivation leads to an increase in the rates of memory traces reproduction in young mice. The animals from the “SD young” group were the first to enter the zone where the platform used to be located, and 70% of them swam straight to the target (direct searching strategy). The mice from the “SD adult” group are mostly preferred active and chaotic searching strategy (Supplementary Figure 2). Moreover, the active searching strategy was accompanied by sharp turns and reversing the direction that point to the animal's loss of concentration. The decrease in synaptic plasticity of the adult brain cortex against the background of chronic sleep deprivation influence could be assumed. Therefore, the next stage of the study aims to assess the features of morphological changes in mice's brain cortex after the modeled chronic stress.

### Morphological changes in mice brain cortex under sleep deprivation influence

**Figure 5:**
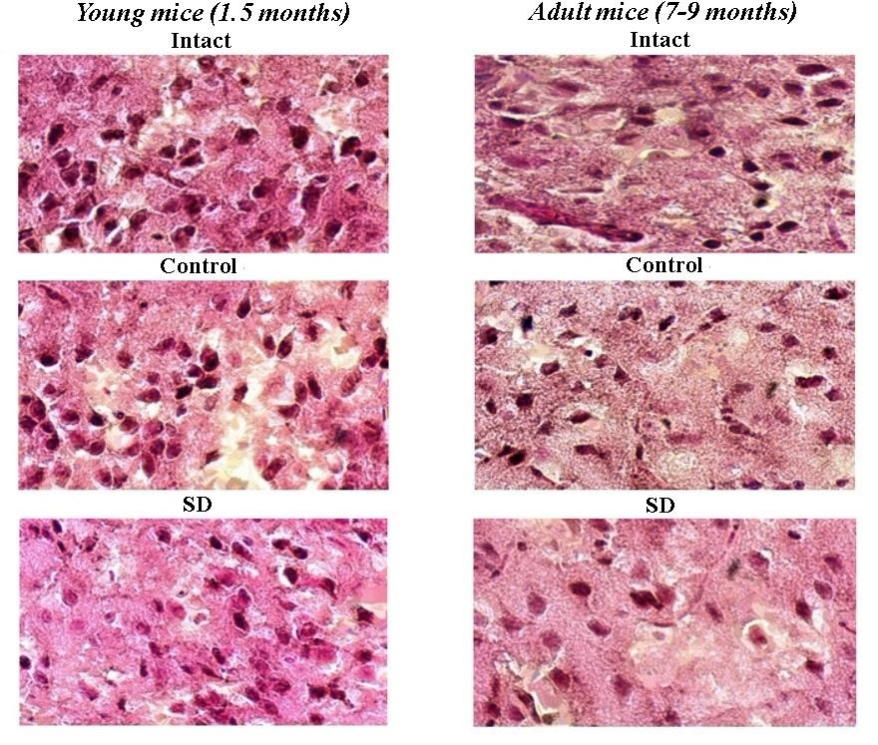
**Representative images of histological samples of murine brain cortex after sleep deprivation modelling.** Hematoxylin-eosin staining, magnification х20.

Histological studies revealed no significant morphological changes of the brain cortex in response to training activity in the Morris water maze. The internal granular layer of the “Intact” and “Control” groups was represented by large neurons (Figure 5). The cells in most cases were pyramidal and stellate in shape; only single rounded and oval cells were observed in the fields of view. The nuclei had a well-defined structure with a rounded and oval shape and occupied, on average, half of the cell volume. The cytoplasm had a regular structure without coarse inclusions. The vessels were not damaged. However, it is interesting to note a tendency towards contraction and branching of neuronal outgrowths in adult animals. Moreover, the average diameter of brain neurons of adult mice was decreased relative to the parameters of young mice (“Intact young” 5.5 ± 0.2 μm; “Control young” 5.3 ± 0.6 μm; “Intact adult” 4.2 ± 0.5 μm; “Control adult” 4.1 ± 0.3 μm).

Sleep deprivation modelling caused morphological changes in nerve cells. In the “SD” group, the average diameter of the soma was decreased (“SD young” 4.14 ± 0.4 μm, “SD adult” 3.9± 0.4 μm); the neurons became more elongated and less branched that point to signs of the degradation of synaptic contacts. At the same time, the appearance of new neurons was observed. This effect was more pronounced in young mice, where the number of new cells in 10 fields of view was in 2–3 times higher relative to those in adult mice (“SD young” 4.1 ± 0.94 μm, “SD adult” 0.9 ± 0.35 μm). It could be assumed that young animals have an increased adaptive potential for substantial restructuring in a sleep-awake cycle.

### Mitochondrial functional activity in the mice brain under sleep deprivation influence

As a powerful stress factor, long-term sleep deprivation can trigger the cell death processes in which mitochondria are often actively involved [53, 54]. Therefore, assessment of the mitochondrial functional activity can serve as a critical parameter of brain cells adaptation to stress conditions.

Our studies revealed a tendency to decrease the main parameters of mitochondrial functional activity in young mice from the “Control” group, which is probably related to their activity in passing training courses in the Morris water maze (Table 2). Spatial learning, combined with the long-term influence of sleep deprivation, promptly adapted the mitochondrial brain cells' apparatus by activating cellular respiration through the production of molecular components in actively working mitochondria. No significant differences in the main parameters of mitochondrial functional state in mice brain of the “SD” group were found relative to the values of the “Intact” and “Control” groups.

**Table 2:** The main parameters of mitochondrial functional activity in the mice brain after sleep deprivation modeling (M±SEM, pmol/(s*mL)).

**(A) Young mice (1.5 months).**						
**Experimental groups**	**Basal of oxygen consumption rate (V4)**	**ADP-stimulated respiration (V3)**	**Inhibition of NADH dehydrogenase (inhibition of respiratory chain complex I)**	**Activation of an alternate pathway for the respiratory chain (activation of respiratory chain complex II)**	**Proton leakage**	**Mitochondrial activity in V3/V4 state**
Intact	68.5±2.5	200.0±39	17.0±3.0	147.0±19	41±4.26	2.95±0.65
Control	37.8±8.1	116.2±21.5	8.7±1.4	80.7±12, 1	52±5.26	3.32±0.7
SD	63.3±8.0	195.5±24.8	10.5±1.3	120±14.5	41.25±3.52	3.15±0.3

**Table:** 

**(B) Adult mice (7-9 months).**						
**Experimental groups**	**Basal of oxygen consumption rate (V4)**	**ADP-stimulated respiration (V3)**	**Inhibition of NADH dehydrogenase (inhibition of respiratory chain complex I)**	**Activation of an alternate pathway for the respiratory chain (activation of respiratory chain complex II)**	**Proton leakage**	**Mitochondrial activity in V3/V4 state**
Intact	28.8±3.5	116.5±21.5	6.7±1.1	81.8±21.0	37±4.69	4.46±0.97
Control	28.2±3.0	112.7±20.3	5.6±0.8	81.8±9.7	39.22±4.89	4.3±0.78
SD	34.3±4.5**&**	120.9±20.8**&**	6.75±0.7**&**	86.3±11.5	40.12±3.01	3.77±0.6

& - versus “SD” of the adjacent age group, p≤0.05, the Wilcoxon T-test.

However, it should be noted that aging potentially leads to decreasing the work of the mitochondrial respiratory chain and the intensity of oxidative phosphorylation. A downward tendency in the basal of oxygen consumption rate and mitochondrial respiration activity in inhibition of respiratory chain complex I as well as during activation of respiratory chain complex II in the adult mice from all groups were observed (Table 2). The reduced activity of respiratory chain complexes I and II could potentially serve as a trigger for decreasing the animal resistance to stressogenic effects.

### Assessment of molecular markers of brain adaptation and aging processes in response to sleep deprivation influence

**Figure 6:**
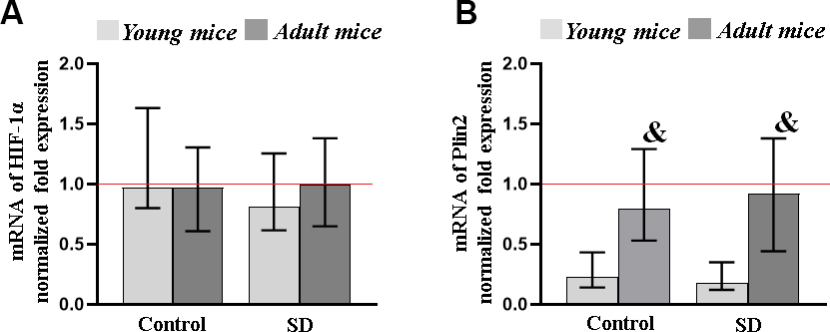
The level of transcription factor HIF-1α (**A**) and PLIN2 (**B**) genes expression in the mice brain after sleep deprivation modeling. Data are normalized to the reference gene (Oaz1), * - versus “SD young”, p≤0.05, the Kruskal-Wallis test.

Next, using the RT-qPCR method, we analyzed the level of gene expression of α-subunit of hypoxia-inducible factor HIF1 (HIF1α) and perilipin 2 (PLIN2) in the mice brain after sleep deprivation modeling (Figure 6). HIF1α is one of the key participants that control oxygen homeostasis and adaptive processes in the brain under the influence of various stress-factors [55, 56]. PLIN2 is a protein involved in lipid storage and metabolism in non-adipose tissues which accumulation is regarded as a potential marker of age-related processes in the organism.

The studies revealed no significant changes in HIF1α expression level in both young and adult mice subjected to sleep deprivation modeling. This suggests that HIF1α is involved in short-term rather than long-term adaptive processes to the effects of chronic stress.

The influence of sleep deprivation modeling also did not lead to significant changes in PLIN2 expression level in brain cells. However, PLIN2 alterations were depended on the animals’ age. The PLIN2 expression level in the brain of mice of 7-9 of age from both the “Control” and “SD” group was 1.04 ± 0.19 and 1.05 ± 0.78, respectively, which significantly exceeded the values of young specimens. Thus, our data are consistent with the previous studies showing the importance of PLIN2 identification as a marker for assessing age-related brain changes.

### Alterations in the level of DNA methylations under sleep deprivation influence

To assess the changes in cellular metabolic rate and physiological state of mice and the renewal capacity of cells during aging and sleep deprivation influence, a method for registration of DNA chain damages was used.

**Figure 7:**
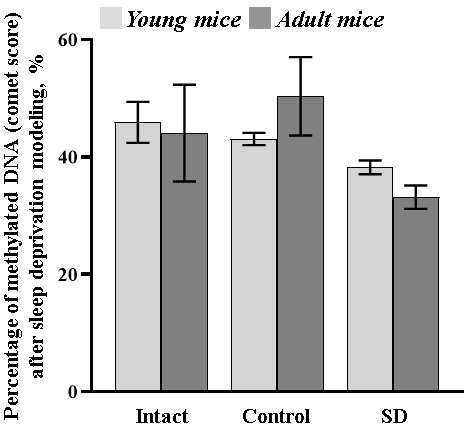
**The percentage of methylated DNA (comet score) in the blood samples of female C57BL/6 mice after sleep deprivation modeling.** No statistical differences between groups, p≤0.05, the Mann-Whitney test.

The data showed a downward tendency in the percentage of the methylated DNA in both young (comet score: “SD young” 38.22 ± 1.17%) and adult (comet score: “SD adult” 33.14 ± 1.99%) mice exposed to sleep deprivation modeling (Figure 7).

It could be assumed that a decrease in the level of DNA methylation aggravated with age implies the emerging risk of depletion in the adaptive capacity of the organism, as was shown in particular in our comprehensive study.

## DISCUSSION

In view of the steady increase in life expectancy of the world population, research into the mechanisms of aging at different organization levels, and the development of a sound strategy for ensuring healthy aging, are becoming more important. Aging is a gradual multifactorial process leading to a loss of body function, development of age-related diseases and pathologies accompanied by aging processes. At present, there is no generally accepted concept about aging as well as a list of stress-factors and molecular markers that affect the rate of this process development. Insomnia is considered one of the potential triggers of accelerated aging [57–59]. The frequency of registered sleep-wake cycle complaints increases with age and correlates with the quality of life of elderly people. Nevertheless, whether insomnia is actually an age-associated process or whether it acts as an independent stress-factor that activates pathological processes, including neurodegeneration, remains controversial.

Natural physiological changes in circadian rhythm influence many older people to go to bed and wake up earlier; it contributes to the deterioration of sleep quality and reduction of sleep duration. Transient insomnia lasts up to 7 days and is caused by severe short-term stress. This stress is triggered by a circadian rhythm disturbance and is mediated by the development of psychophysical disorders, which might be irreversible. It can lead to the development of chronic insomnia accompanied by subsequent difficulties in falling asleep. The additional impact of other stress factors can aggravate sleep-wake cycle disturbances and stimulate the development of age-related pathologies [16, 23].

In this comprehensive study, we studied the effects of long-term sleep deprivation on the physiological state of female mice of different ages, their mnestic and cognitive abilities, and evaluated the morpho-functional characteristics and adaptive capabilities of brain cells after the modeled stress exposure. The sex of the experimental animals was chosen based on the previously established greater susceptibilities of females to sleep-wake cycle disorders [35, 60].

Sleep deprivation is known to reduce physical activity and significantly impacts the behavior and emotional state of humans and animals [5]. Our study showed that long-term sleep deprivation modeling leads to pronounced changes in the eating behavior of mice characterized by the increased food intake during simulated stress. The observed alterations presumably related to changes in melatonin metabolism [50], as well as increased levels of cortisol, phenylalanine and aspartic acid, which are able to suppress the release of several neurotransmitters in the brain [51, 61]. Lack of sleep can also lead to increased production of the appetite-stimulating hormone ghrelin [62, 63]. We showed that against the background of the increased food intake, adult mice significantly gained weight, while young mice, on the contrary, lost weight. Previous studies demonstrated that changes in weight characteristics are independent of the period of the sleep-wake cycle and the duration of sleep deprivation [5, 64–66] that points to the age-mediated nature of our established data. Analysis of behavioral reactions in the “Open Field” test revealed an increase in locomotor and exploratory activity and an unstable emotional state in both young and adult mice after sleep deprivation modeling. It can be assumed that such behavioral changes in response to new stress (an open, brightly lit space) are associated with gradually cumulative response to long-term sleep deprivation and related to the hyperactivation of the nervous system. Young mice respond to stress more actively, probably due to their increased adaptive capacity relative to adult animals [61, 67].

As a powerful stress-factor, sleep deprivation can lead to impaired cognitive function, decreased ability to correctly set tasks and their high speed and accuracy of execution, which is mediated by pronounced changes in brain plasticity [68, 69]. Early studies point to sleep’s essential role in short-term memory reorganization mechanisms into the long-term once [66, 70]. Our study showed that in a normal state, the adult mice were less amenable to spatial learning and spent more time reaching the target in the Morris water maze training sessions than young mice. In conditions of circadian rhythm disturbance, the activation of mnestic functions in young mice was noted. The long-term memory retention test parameters in the “SD young” group exceeded those of the sleeping mice. Moreover, the young mice chose a straightforward searching strategy to reach the goal, and the percentage of negative results was minimal. The influence of sleep deprivation leads to severe violations in the mnestic functions of adult mice. The specimens from the “SD adult” group switching their tactics in the Morris water maze into the active and chaotic movement trajectory with an increased number of negative attempts. Our data point to age-dependent changes in brain plasticity, which obviously should correlate with changes in the morpho-functional characteristics of neuron-glial networks. Previous studies emphasized the enhanced cortical signaling in response to a shift in the sleep-wake cycle [71]. In the long-term sleep deprivation state, activation of microglia and increased area of presynaptic endings in different age groups were shown [72, 73]. In histological studies, we demonstrated remarkable morphological changes in the murine brain cortex after the long-term sleep deprivation modelling, characterized by alterations in shape and decreased size of the neuronal soma as well as reduction of neuronal outgrowths branching. However, against the background of these changes, the appearance of new cells in the young brain cortex was observed, which predisposes this age group of mice to increased stress resistance and intensive recovery of the metabolism during the short sleep period.

Next, we considered the changes in the intensity of brain mitochondrial functional activity and the expression level of hypoxia-inducible factor HIF1α as potential markers of the degree of resistance and intensity of adaptive processes in brain cells to sleep deprivation effects. These are the most sensitive nodes to stressogenic effects, as they are key participants in energy metabolism and the control of oxygen homeostasis [55, 56, 74].

Our studies revealed that the main parameters of mitochondrial functional activity and the level of HIF1α expression in brain cells during sleep deprivation remained at the level of values registered from animals with a normal sleep-wake cycle. However, it is interesting to note a tendency to decrease in the mitochondrial respiratory chain work and intensity of oxidative phosphorylation in adult mice. Since these changes can further lead to the activation of reactive oxygen species accumulation and the development of oxidative stress [75], they can potentially be considered an early diagnostic age-associated marker of the decreased adaptive potential of brain cells.

We also attempted to analyze the effect of long-term sleep deprivation on the activation of accelerated aging processes. For this purpose, we examined the expression level of PLIN2, one of the potential aging markers [55], and assessed the level of DNA methylation in mice of different age groups. Sleep deprivation modelling did not cause significant changes in PLIN2 expression and DNA methylation in 1.5-month-old female mice that points to that the adaptive capabilities of animals from this age group allow preserving the resistance to accelerated aging processes. In adult mice, against the background of increased PLIN2 levels relative to young individuals, sleep deprivation influence leads to a downward tendency in DNA methylation levels. Epigenetic changes, including DNA methylation, are an essential component of the complex aging process. Since global hypomethylation of DNA is known to occur with age [76–79], we can assume that longer sleep deprivation influence will result in disruption of adaptation and activation of accelerated aging and neurodegeneration processes.

In summary, our findings point to the age-related adaptive capacity of female mice to the long-term sleep deprivation influence. For young mice (1.5 months of age), the modeled sleep deprivation acts as a stress factor leading to weight loss against the background of increased food intake, the activation of animals locomotor and exploratory activity, their mnestic functions, as well as molecular and cellular adaptive processes ensuring animal resistance both to stress and risk of accelerated aging development. Sleep-wake cycle disruption in adult female mice (7-9 months of age) is accompanied by an increase in body weight against the background of active food intake, increased locomotor and exploratory activity, gross disturbances in mnestic functions, and decreased adaptive capacity of brain cells, that potentially increasing the risk of pathological reactions and neurodegenerative processes with more prolonged sleep deprivation influence or other stress factor exposure.

## MATERIALS AND METHODS

### Ethics statement

The experiments were carried out on female C57BL/6 mice (n=77). All experimental protocols used in the study were approved by the Bioethics Committee of Lobachevsky University and carried out in accordance with Act708n (23.08.2010) of the Russian Federation National Ministry of Public Health, which states the rules of laboratory practice for the care and use of laboratory animals, and the Council Directive 2010/63 EU of the European Parliament (September 22, 2010) on the protection of animals used for scientific purposes.

### Experimental design

Female C57BL/6 mice were divided into the following groups: 1) Intact animals (n=13); 2) Control – the animals undergoing training in the Morris water maze without sleep deprivation modeling (n=32); 3) SD – the animals undergoing training in the Morris water maze followed by sleep deprivation modeling (n=32). Each group consisted of 1.5 (young) and 7-9 (adult) months mice.

**Figure 8:**
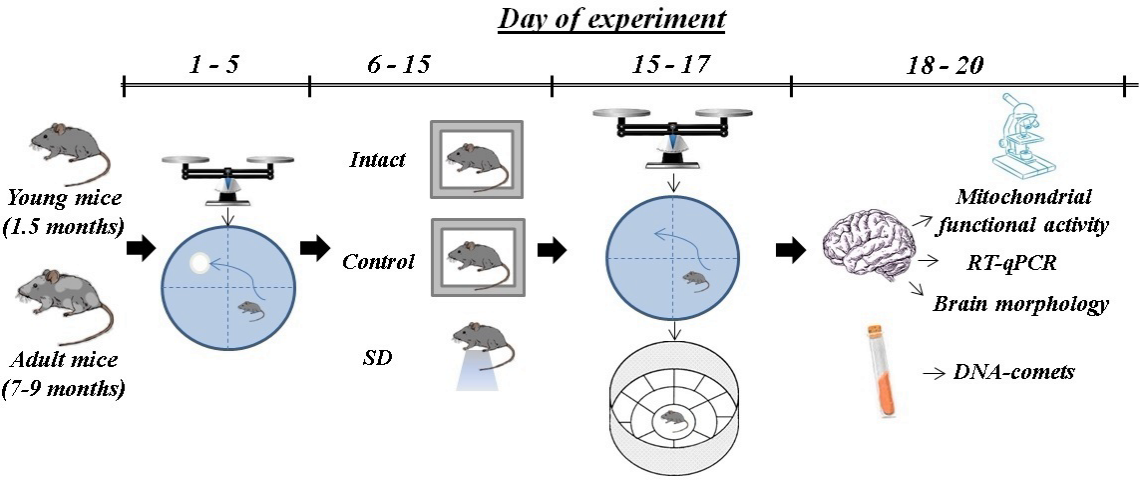
Scheme of the experiment.

Before starting the experiment, mice were weighed and then tested in the Morris water maze setup (Figure 8). As the Morris water maze test is associated with considerable physical activity, the “Intact” group was added to the experiment to evaluate its contribution to the studied parameters. The next day after the last training, sleep deprivation modeling was performed. Afterwards, the mice were re-weighed, and their general locomotor and orienting-exploratory activity were assessed in the “Open Field” setup. Besides, we analyzed the long-term memory retention in mice by testing them in the Morris water maze without a platform for 1 min. Then the murine brains were collected for further registration of mitochondrial functional activity, the HIF-1α (Hif1) and Plin2 genes expression level (RT-qPCR analysis) and histological analysis. The level of DNA methylations was assessed in the blood samples.

### Sleep deprivation model

Animals were stressed using the flowerpot method, first used by M. Jouvet and colleagues to modulate deprivation of REM stage of sleep [49], with modifications. The experiments began daily at 10-11 a.m. A platform (d=3 cm) was located 1-2 cm below the water filling the circular pool. Each animal was placed on the platform and kept awake for 10 hours a day for five days. For the following five days, the mouse spent on the platform for 11 hours a day and additionally received sound signals every hour starting at 5 a.m.

### “Open field” test

The general locomotor and orienting-exploratory activity of the experimental animals were tested in the “Open Field” setup (OpenField LE800S; Panlab Harvard Apparatus, Spain) before and a day after sleep deprivation modeling. Behavioral reactions were registered by a Sony SSC-G118 (Japan) camera for 5 min. The obtained data were analyzed in a Smart 3.0.03 software program (Panlab Harvard Apparatus, Spain; Stoelting, USA). The following behavioral reactions of mice were analyzed: 1) an orienting-exploratory activity (the number of squares crossed around the arena perimeter, the number of peeks in the holes, the number of upright postures); 2) emotional state (the number of squares crossed in the arena center, the residence time in the arena center); 3) passive reaction fear (frequency of grooming acts and the number of boluses).

### Morris water maze test

To assess spatial learning and working memory of mice, the Morris Water Maze test with modifications was used [80, 81]. The test was conducted in a green circular pool (d = 900 mm, h = 500 mm) filled with opaque water (24±1° C) in which a white Plexiglas platform (d = 7.6 cm) was placed 1-2 cm below the water surface. The mice were trained for 5 days. Each session was 3 times for 60 min. The animal was trained to find the platform by an external visual landmark by placing it from different pool sides. If the mouse could not find the platform by the end of session time or jumped from the platform, it was forced to stand on it. The long-term memory retention was assessed by testing animals in a pool without a platform for 1 min. Data collection was automated using Smart Tracking (Panlab Harvard Apparatus, Spain; Stoelting, USA). The time of staying in a zone where the platform was previously located, and the type of strategy for searching the platform were assessed.

### Registration of brain mitochondrial functional activity

Brain mitochondria were isolated using the standard differential centrifugation method [82]. Oxygen consumption by the isolated mitochondria was registered polarographically using a high-resolution respirometer Oxygraph-2k (Oroboros, Austria) in a closed chamber at constant stirring and controlled temperature (37° C). Incubation medium for mitochondria contained 120 КCl mM, 5 NaH2PO4 mM, 10 HEPES, 5 mM glutamate, 5 mM malate, and 14 mM MgCl2 (pH 7.4). The concentration of mitochondrial protein in a chamber measured by the Bradford method was 0.5 mg/ml.

**Figure 9:**
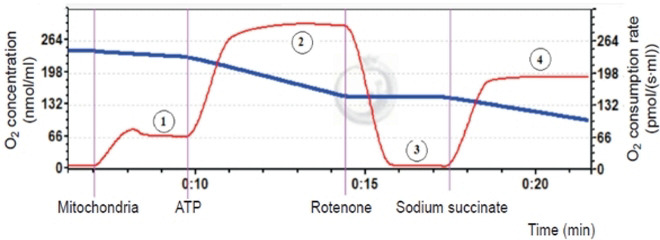
**A typical example of recordings the oxygen consumption rate by brain mitochondria.** Respiratory chain components were added sequentially to the incubation medium containing mitochondrial suspension: (1) oxygen consumption rate in the presence of a high concentration of complex I substrates: 5 mM glutamate and 5 mM malate (electron transfer from NADH hydrogen atoms to respiratory chain enzymes); (2) stimulation of the oxidative phosphorylation of the mitochondrial respiratory chain by 5 mM ADP (the oxidation process of reduced NADH equivalents by the respiratory chain enzymes followed by the ATP synthesis); (3) inhibition of the complex I work with 0.5 μM rotenone solution (electron transfer blockade in complex I from the iron-sulphur cluster to the oxidized ubichinon); (4) succinate-dependent pathway of substrate oxidation (application of complex II substrate (succinate)).

The functional state of the mitochondrial respiratory chain was assessed according to the following parameters (Figure 9): a) V4 — the rate of oxygen consumption by the mitochondria at high content of substrates 5 mM glutamate and 5 mM malate (substrates of complex I) in the incubation medium; b) V3 — oxidative phosphorylation rate in V4 conditions supplemented with 5 mM adenosine diphosphate (ADP). The intensity of complex II of the respiratory chain was assessed after complex I inhibition by 0.5 μM rotenone and complex II stimulation with 10 mM sodium succinate.

### RNA extraction and RT-qPCR

The expression level of HIF-1α (Hif1) and Plin2 genes was analyzed by quantitative real-time PCR. Total RNA was extracted from the mouse brain using ExtractRNA (Evrogen, Moscow, Russia). Then cDNA was synthesized using the MMLV reverse transcriptase kit and a random primer (Evrogen, Moscow, Russia). Amplification was carried out in real-time PCR using the qPCRmix-HS SYBR kit (Evrogen, Moscow, Russia) on an Applied Biosystems 7500 RT-PCR amplifier. The following pairs of primers were used:

Hif1a_fw1 - 5'-GCAATTCTCCAAGCCCTCCAAG-3';

Hif1a_rv1 - 5'-TTCATCAGTGGTGGCAGTTGTG-3';

Plin2_fw-5'-AAGCTGGAGCCACAAATTGC-3';

Plin2_rv - 5'-TGGCACTGGCAACAATCTCG-3';

Oaz1_fw - 5'-AAGGACAGTTTTGCAGCTCTCC-3';

Oaz1_rv - 5'-TCTGTCCTCACGGTTCTTGGG-3';

Data processing was carried out using the ΔΔCt method and a reference sample in which the target gene level was taken as a unit. Normalization was performed relative to the reference gene (Oaz1).

### Brain morphology assessment

For histological studies, the brains isolated from experimental animals were fixed in a 10% formalin solution for 24 h at room temperature. For the next 24 h, the samples were placed in a 15% sucrose solution followed by incubation in a 30% sucrose solution for 24-48 h. After that, the brain was placed on a platform of freezing sliding cryostat Leica CM1520 (Leica, Germany) and gradually filling with cryogel (Leica, Germany) at 30° C. Next, the sample was cut into 10 μm thin coronal sections. Every fifth section was mounted on a glass slide and dried in the air for 24 h. The sections were then stained by the hematoxylin-eosin method [83]. Then, the sections were dehydrated in alcohols of upward concentration, purified in xylenes and embedded in a mounting medium (Consul-Mount, USA). The samples were examined using a Zeiss Primo Star light microscope (Germany) with integrated an Axio CamMRc camera (Zeiss, Germany).

### DNA-comets

Analysis of DNA methylation was performed using a comet test developed by Wentzel and colleagues [84] with some modifications.

Aliquots of blood samples were mixed with 0.5% agarose (low melting point) and then placed on a glass slide pre-treated with 1% agarose (high melting point). The cells were stored in a buffer (2.5 M NaCl, 0.4 mM Na_4_EDTA, 10% DMSO, 1% Triton X-100 (pH 8)) at 4° C overnight. Next, the slides were washed in a buffer containing 10 mM NaCl, 2 mM EDTA, 10 mM Tris-HCl, 1 mM B-mercaptoethanol (pH 7.9). Each slide was then treated with HpaII or MspI enzyme mixture (15 U/mL, CutSmart Buffer), covered with a coverslip and incubated in a humid chamber for an hour at 37° C. Electrophoresis was carried out in a 1.0 V/cm, 300 mA, 4° C regimens for 45 min. The slides were then fixed in ethanol, stained with DAPI (1 μg/ml) and examined in an LSM 800 confocal microscope (Zeiss, Germany). Electrophoresis leads to the formation of comet-like structures, and using fluorescence microscopy, the number of DNA breaks can be analyzed by the intensity of brightness of the comet tail [85].

For each sample, 100 cells were counted, and the percentage of DNA in comet tails (Tail DNA, %) was determined. The percentage of CpG dinucleotides methylation was calculated using CometScore 2.0 software and the following formula:

$$100-\frac{\%T\;Hpall}{\%T\;Mspl}\times 100$$ (1)

where % T HpaII and % T MspI - are the average percentage of tails in a slide treated with the corresponding enzyme.

### Statistical data analysis

The quantified data are presented as the mean ± standard error of the mean (SEM). GraphPad Prism (v.9.0) was used for statistical analysis. The Shapiro-Wilk test was used for normal distribution analysis. Differences between two independent groups were assessed by using the Mann-Whitney test. In order to determine the equality of the mean values in the two groups, the Wilcoxon T-test was applied. The Kruskal-Wallis test was used to assess the equality of medians of several groups. Differences between groups were considered significant if the corresponding p-value was less than 0.05.

## Supplementary Material

Supplementary Figures

Supplementary Table 1
