# Supplementary material for: Features of age-related response to sleep deprivation: in vivo experimental studies
Source: Aging (Albany NY). 2021 Jul 28;13(15):19108–26. doi: 10.18632/aging.203372 (PMC8386558; doi:10.18632/aging.203372)
Supplement: Supplementary Figures [file aging-13-203372-s001.pdf]

SUPPLEMENTARY FIGURES

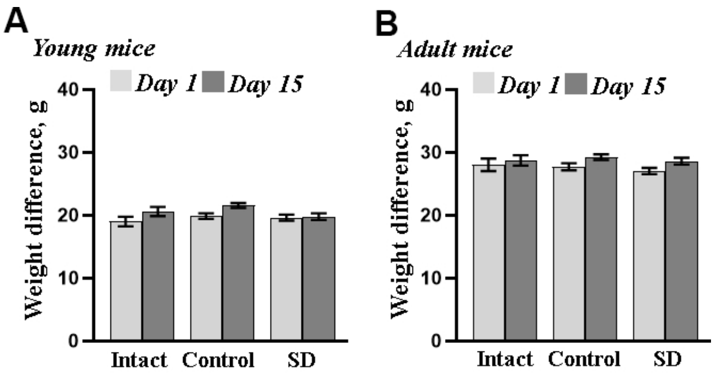

Supplementary Figure 1. Body weight of female C57BL/6 mice at the beginning (day 1) and by end (day 15) of sleep deprivation modeling. (**A**) - Young mice (1.5 months); (**B**) - Adult mice (7-9 months). No statistical differences between groups, p≤0.05, the Wilcoxon T-test.

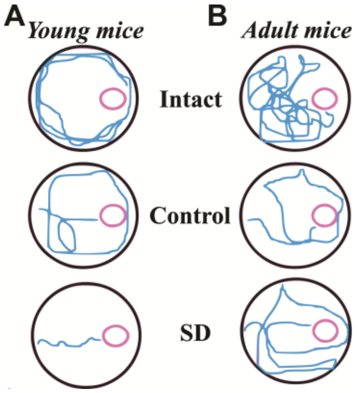

Supplementary Figure 2. Main target searching strategies of female C57BL/6 mice during long-term memory retention test in the Morris water maze after sleep deprivation modeling. (**A**) Young mice (1.5 months); (**B**) Adult mice (7-9 months).
