# Supplementary material for: Features of age-related response to sleep deprivation: in vivo experimental studies
Source: Aging (Albany NY). 2021 Jul 28;13(15):19108–26. doi: 10.18632/aging.203372 (PMC8386558; doi:10.18632/aging.203372)
Supplement: Supplementary Table 1 [file aging-13-203372-s002.pdf]

SUPPLEMENTARY TABLE

Supplementary Table 1. Parameters of behavioral reactions of female C57BL/6 mice on the «Open field» test day after the end of sleep deprivation.

(A) Young mice (1.5 months).

Experimental groups	Acts of defecation	Acts of urination	Acts of grooming		Number of upright postures
			Time of short grooming, s	long	
Intact	0.16±0.16	0.16±0.16	2.13±0.94	0.16±0.16	8±1.87
Control	1.8±0.53*	0.3±0.15	9±2.27§	0.5±0.3	4.5±7.1
SD	1.1±0.58	0	2±0.74 ˠ	0.4±0.22	4.3±1.01

(B) Adult mice (7-9 months).

Experimental groups	Acts of defecation	Acts of urination	Grooming		Number of upright postures
			Time of short grooming, s	Number of long acts of grooming	
Intact	0.16±0.16ˠ	0.16±0.16	2.5±1.05	1.16±0.98	12.83±1.97
Control	1.22±0.32	0.13±0.07	9.54±2.51 *§	0.68±0.28	6.59±1.0
SD	0.28±0.17ˠ	0	3.38±0,82 #ˠ	0.38±0.17	10.19±1.26 #&

*- versus "Intact", # - versus "Control", § - versus "Intact" of the adjacent age group, ˠ - versus "Control" of the adjacent age group, & - versus "SD" of the adjacent age group, p≤0.05, the Mann-Whitney test.
